# Clinicopathological characteristics of early gastric cancer associated with autoimmune gastritis

**DOI:** 10.1002/jgh3.12656

**Published:** 2021-09-16

**Authors:** Shinji Kitamura, Naoki Muguruma, Koichi Okamoto, Kaizo Kagemoto, Yoshifumi Kida, Yasuhiro Mitsui, Hiroyuki Ueda, Tomoyuki Kawaguchi, Hiroshi Miyamoto, Yasushi Sato, Rika Aoki, Joji Shunto, Yoshimi Bando, Tetsuji Takayama

**Affiliations:** ^1^ Department of Gastroenterology and Oncology Tokushima University Graduate School of Biomedical Sciences Tokushima Japan; ^2^ Tokushima Health Screening Center Tokushima Japan; ^3^ Shunto Clinic Tokushima Japan; ^4^ Division of Pathology Tokushima University Hospital Tokushima Japan

**Keywords:** endoscopy, gastric cancer, stomach

## Abstract

**Background:**

Autoimmune gastritis is known to be associated with neoplastic lesions but the relationship between autoimmunity and tumorigenesis have not been sufficiently clarified. The aim of this study is to assess the clinicopathological characteristics of gastric cancer cases associated with autoimmune gastritis.

**Methods:**

A total of 24 patients diagnosed as early gastric cancer with autoimmune gastritis were registered. Chart reviews with the data including age, gender, state of *Helicobacter pylori* infection, comorbidity, and concomitant gastric diseases were conducted. As for the characteristics of gastric cancer, location, size, morphological type, histopathology, invasion depth, and the presence of metachronous or simultaneous lesion were assessed. These data from autoimmune gastritis group were compared with those from 301 patients of early gastric cancer as a control group.

**Results:**

The gastric cancer associated with autoimmune gastritis was located in the upper, middle, and lower parts in 28.1%, 53.1%, and 18.8%, respectively. The morphological types are as follows: 0‐I, 9.4%; 0‐IIa, 28.1%; 0‐IIb, 15.6%; 0‐IIc, 46.9%; and 0‐III, 0.0%. The mean tumor size was 21.8 mm. While 90.6% were confined to the mucosa, 9.4% showed submucosal invasion. The histological classifications are as follows: tub1, 50.0%; tub2, 15.6%; pap, 21.9%; sig, 9.4%; and por, 3.1%. More numbers of female, protruded types, larger tumor size, papillary tumor, and that in the upper location were observed in autoimmune gastritis group compared to control group.

**Conclusion:**

Early gastric cancer associated with autoimmune gastritis demonstrated different characteristics from those without autoimmune gastritis including variety of tumor morphologies and histological types with female dominancy.

## Graphical abstract

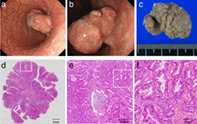

Autoimmune gastritis is known to be associated with neoplastic lesions but the relationship between autoimmunity and tumorigenesis have not been sufficiently clarified. This study assessed the clinicopathological characteristics of early gastric cancer cases associated with autoimmune gastritis. Early gastric cancer associated with autoimmune gastritis demonstrated different characteristics from those without autoimmune gastritis including variety of tumor morphologies and histological types.

## Introduction

Gastric cancer incidence and mortality have markedly declined over the past decades mainly due to the decrease of *Helicobacter pylori* (*H. pylori*) infection.^1^, ^2^ However, gastric cancer still ranks the third leading cause of cancer death worldwide and other risk factors except for *H. pylori* infection have become interested. Autoimmune conditions are important causes of chronic inflammation which could lead to cancer development in various organs and appear to have increased in prevalence recently.^3^ Moreover, a systematic review and meta‐analysis revealed that the wide range of autoimmune diseases including Rheumatoid and Celiac disease are associated with gastric cancer.^4^ Autoimmune gastritis (AIG) is a type of chronic gastritis characterized by production of antibodies against parietal cell and intrinsic factor, resulting in marked mucosal atrophy of gastric body and suppression of acid secretion.^5^, ^6^ While the certain prevalence of AIG is yet unclear because the early diagnosis of this entity is difficult even with a gastric biopsy, it is presumed that there is an unreported link between AIG and various autoimmune diseases.^7^ AIG shows possible progression to impaired vitamin B12 absorption and pernicious anemia at a later stage and is also known to be associated with neoplastic lesions such as gastric carcinoma and neuroendocrine tumor,^8^, ^9^, ^10^, ^11^, ^12^ however, the relationship between autoimmunity and tumorigenesis have not been sufficiently clarified. In the era of eradicated or negative *H. pylori* infection, AIG has been paid more attention as an important background of gastric cancer development. Given these situations, establishment of an optimal period of surveillance endoscopy for AIG is required.^13^ The aim of this study is to assess the clinicopathological characteristics of gastric cancer cases associated with AIG comparing to those without AIG.

## Materials and methods

### Patients and methods

**Figure 1:**
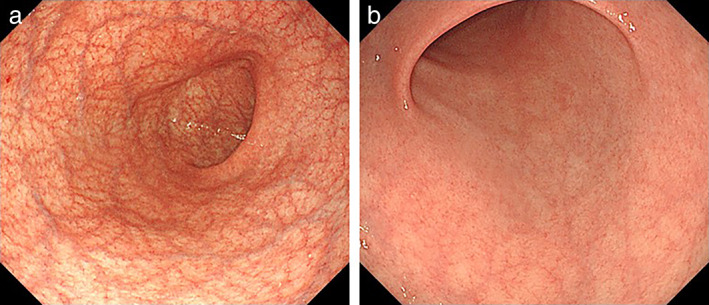
Endoscopic appearance of autoimmune gastritis. (a) A view of the corpus showing marked vascular visibility both on the lesser curvature and the entire greater curvature. (b) A view of the antrum showing no atrophic pattern.

**Figure 2:**
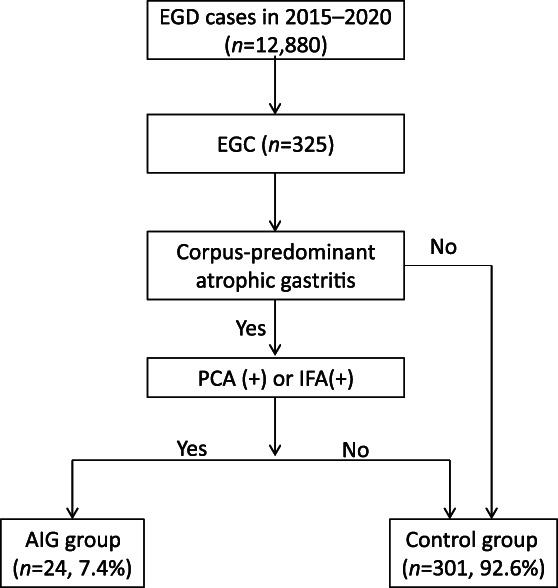
Flowchart of selection of patients for this study. AIG, autoimmune gastritis; EGC, early gastric cancer; EGD, esophagastroduodenoscopy; IFA, anti‐internal factor antibody; PCA, anti‐parietal cell antibody.

A retrospective observational study was performed at Tokushima University Hospital (Tokushima, Japan) between 2015 and 2020. Endoscopic procedure was carried out as further assessment to determine the treatment strategy (endoscopic resection or surgical treatment) in all cases. All methods were carried out in an accordance with relevant guidelines and regulations. In the present study, we defined AIG as cases based on characteristic endoscopic appearances such as significant corpus‐predominant atrophy (Fig. 1)^14^ and positive autoantibodies, either antigastric wall cell antibodies (PCA) or anti‐internal factor antibodies (IFA). Chart reviews with the data including gender, age, and state of *H. pylori* infection, comorbidity, concomitant gastric diseases, and serum gastrin level were conducted. As for the characteristics of gastric cancer, tumor location, morphological type, tumor size, depth of invasion, histopathological type, and the presence of metachronous or simultaneous lesion were conducted. These data from the AIG group were compared with those from 301 cases (320 lesions) of early gastric cancer as a control group after an exclusion of AIG (Fig. 2). Cases with tumors of which central lesion is located proximal to the cardia were excluded. The Institutional Review Board of Tokushima University Hospital provided permission for this study (No. 3480) to conduct chart reviews for each patient and informed consent was obtained from all subjects.

### Tests for antibody

Anti‐PCA were determined by indirect fluorescent antibody tests by using a commercially available kit (Fujirevio Inc., Tokyo, Japan) setting X10 as the cut‐off value. Anti‐IFA were determined by using a commercially available kit (Beckman Coultar, Brea, CA, USA) based on chemiluminescent enzyme immunoassay (CLEIA).

### Determination of *H. pylori* infection

Serum anti‐*H. pylori* IgG antibody was measured for each patient with AIG. When the result was positive, patients were regarded as *H. pylori*‐positive. If the result was negative, additional tests such as urea breath test or rapid urease test were performed and regarded as negative at least two negative tests confirmed.

### Statistical analysis

Continuous variables were expressed as the mean ± standard deviation (SD), while categorical variables were presented as absolute values and percentages. The differences between continuous variables were analyzed using Mann–Whitney U test, and the differences between categorical variables were analyzed using contingency table analysis in Microsoft Excel 2016 software. A *P* value of less than 0.05 was considered to be statistically significant.

## Results

### Patient characteristics

During the period between January 2015 and August 2020, a total of 12 880 esophagogastroduodenoscopies was performed in our hospital. The number of patients diagnosed with early gastric cancer was 325. Among these cases, the patients with corpus‐predominant atrophic gastritis showing positivity to PCA or IFA were 24. A total of 24 patients (7.4%) with 32 lesions were registered consequently (Fig. 2). Fourteen cases were male (58.3%) and the mean age was 70.8 years (range 53–88 years). In AIG‐associated gastric cancer cases, PCA was higher than 40 in 11 the cases (45.8%). IFA were positive in 8 of 24 cases (33.3%). Nine patients (37.5%) were *H. pylori*‐positive and 15 (62.5%) were negative. *H. pylori* eradication therapy had been performed in four cases (16.7%). Other autoimmune diseases including chronic thyroiditis were accompanied in five cases (20.8%) and pernicious anemia was found in four cases (16.7%). Adenomas were found in one case (4.2%), hyperplastic polyps in one case (4.2%), and xanthoma in 16 cases (66.7%). Mean serum gastrin level was 1133 pg./mL (normal range 107–4039 pg./mL). There was no significant correlation between the PCA values of the IFA‐positive and negative groups. These clinical features of the patients with AIG are summarized in Table 1.

**Table 1:** Patients’ characteristics

	*n* = 24 (%)
Sex	
Male	14 (58.3)
Female	10 (41.7)
Age (year, mean, [range])	70.8 [53–88]
PCA	
×10	8 (33.3)
×20	3 (12.5)
×40	8 (33.3)
×80	3 (12.5)
Unknown	2 (8.3)
IFA	
Positive	8 (33.3)
Negative	16 (66.7)
State of *Helicobacter pylori* infection	
Positive	9 (37.5)
Negative	15 (62.5)
Eradication history of *H. pylori*	
Yes	4(16.7)
No	20 (83.3)
Comorbidity (%)	
Autoimmune disease	5 (20.8)
Pernicious anemia	4 (16.7)
Concomitant gastric disease	
Adenoma	1 (4.2)
Hyperplastic polyp	1 (4.2)
Xanthoma	16 (66.7)
Serum gastrin level pg./mL (mean, [range])	1133 [107–4039]

IFA, anti‐internal factor antibody; PCA, anti‐parietal cell antibody.

### Characteristics of the tumor in AIG group

In the AIG group, a total of 32 gastric cancers were diagnosed. This gastric cancer was found to be located in the upper, middle, and lower parts in 28.1%, 53.1%, and 18.8%, respectively. The morphological types of gastric cancer according to the Paris classification are as follows: 0‐I, 9.4%; 0‐IIa, 28.1%; 0‐IIb, 15.6%; 0‐IIc, 46.9%; 0‐III, 0.0%. The mean tumor size was 21.8 mm [range: 7–100]. As for the histological tumor depth, 90.6% of tumors were confined to the mucosa and 9.4% showed submucosal invasion. The proportions of histological classifications among all of the tumors are as follows: tub1, 50.0%; tub2, 15.6%; pap, 21.9%; sig, 9.4%; por, 3.1% (Table 2).

**Table 2:** Characteristics of the tumor in the AIG group

	*n* = 32 (%)
Tumor location	
Upper part	9 (28.1)
Middle part	17 (53.1)
Lower part	6 (18.8)
Morphological type	
I	3 (9.4)
IIa	9 (28.1)
IIb	5 (15.6)
IIc	15 (46.9)
III	0 (0.0)
Tumor size (mm, mean, [range])	21.8 [7–100]
Depth of invasion	
mucosa	29 (90.6)
submucosa	3 (9.4)
Histopathology	
tub1	16 (50.0)
tub2	5 (15.6)
pap	7 (21.9)
sig	3 (9.4)
por	1 (3.1)

pap, papillary adenocarcinoma; por, poorly differentiated tubular adenocarcinoma; sig, signet ring cell carcinoma; tub1, well‐differentiated tubular adenocarcinoma; tub2, moderately differentiated tubular adenocarcinoma.

### Clinicopathological features of gastric cancer between autoimmune gastritis and control groups

The clinicopathological features of the gastric cancer patients with AIG were compared with those without AIG (control group). In terms of gender ratio, the ratio of women was significantly higher in the AIG group (*P* < 0.05). The age at the time of cancer detection was not different from that of the control group. As for location of the gastric cancer, there was a significant difference between AIG and control groups more frequent in the upper or middle parts in the AIG group (*P* < 0.05). There was a significant difference in the morphological type of gastric cancer between the AIG and the control groups. In the AIG group, the ratio of type O‐I and IIa, IIb was higher than the control group (*P* < 0.05). The mean tumor diameter was 21.8 mm in the AIG group and 15.6 mm in the control group. There was a significant difference in the tumor size (*P* < 0.05). There was no significant difference in the depth of invasion between the AIG group and the control group. There was a significant difference in the histological type of cancer (*P* < 0.001). There were more cases of papillary tumor in the AIG group. Undifferentiated cancer with deep submucosal infiltration was also observed in *H. pylori*‐negative AIG cases with no history of eradication. Although there was a tendency to harbor more multiple cancers in the AIG group compared to the control group, there was no statistically significant difference (*P* = 0.288) (Table 3).

**Table 3:** Relationship between clinicopathological features of gastric cancer patients between autoimmune gastritis and control groups

	AIG (*n* = 24, 32 lesion)	Control (*n* = 301, 320 lesion)	*P*‐value
Sex, *n* (%)			0.044
Male	14 (58.3)	231 (76.3)	
Female	10 (41.7)	70 (23.3)	
Age (year, mean, [range])	70.8 [53–88]	71.1 [38–90]	n.s
Location			0.032
Upper	9	46	
Middle	17	150	
Lower	6	124	
Morphological Type			0.022
I	3	7	
IIa	9	70	
IIb	5	26	
IIc	15	217	
Tumor size (mean ± SD)	21.8 ± 19.1	15.6 ± 10.0	0.027
Depth of invasion			n.s
Mucosa	29	283	
Submucosa	3	37	
Histopathology			<0.001
tub1	16	242	
tub2	5	42	
pap	7	2	
sig	3	28	
por	1	5	
Other	0	1	
Multiple gastric cancer	6	47	n.s
Simultaneous	3	17	
Metachronous	3	30	

AIG, autoimmune gastritis; n.s, not significant; pap, papillary adenocarcinoma; por, poorly differentiated tubular adenocarcinoma; sig, signet ring cell carcinoma; tub1, well‐differentiated tubular adenocarcinoma; tub2, moderately differentiated tubular adenocarcinoma.

## Discussion

**Figure 3:**
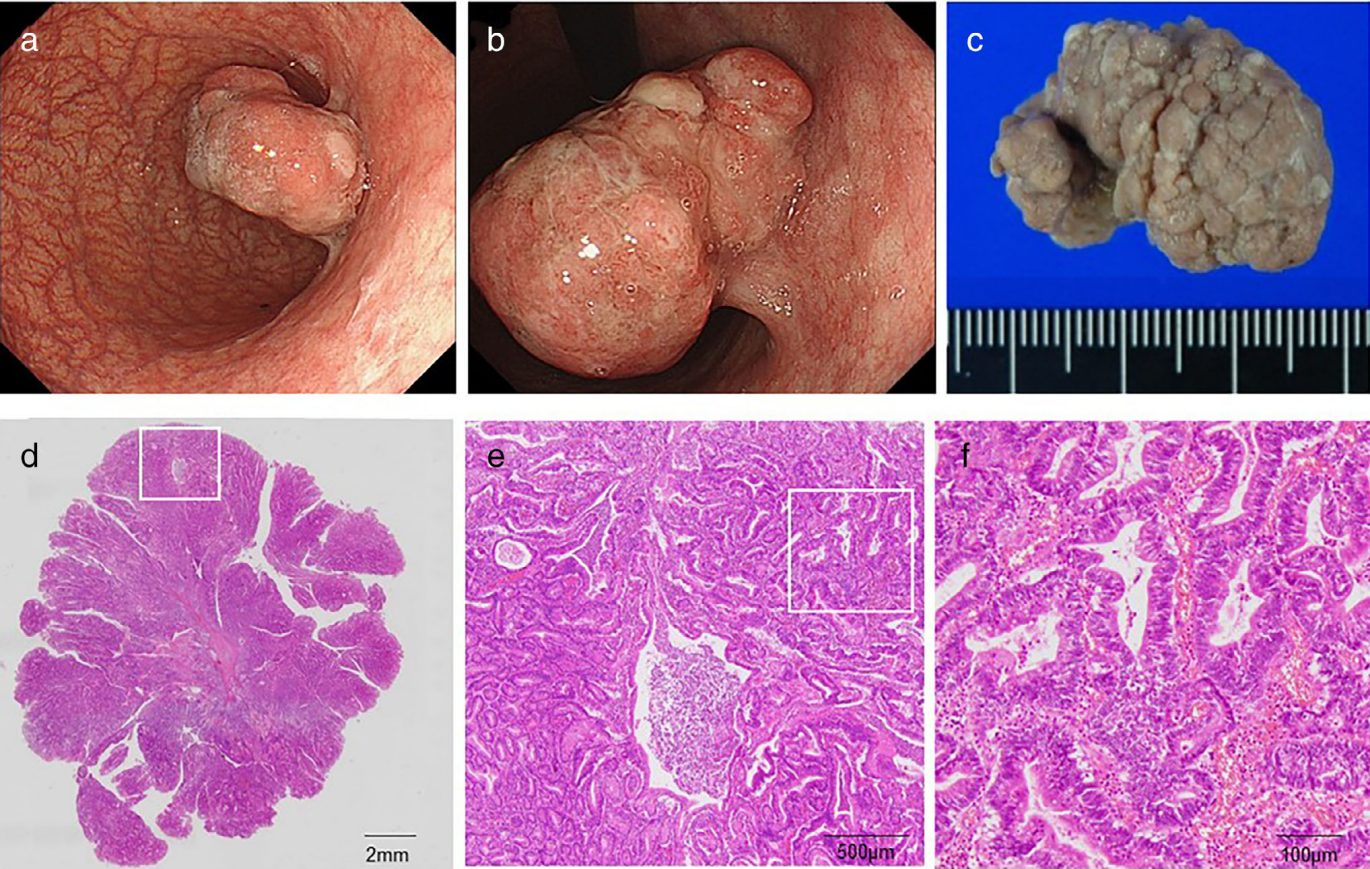
A representative case of adenocarcinoma associated with autoimmune gastritis. (a) A 4 cm‐sized protruded tumor was seen in the middle body, (b) A retroflexed view showing semipedunculated shape and nodular surface pattern. (c) Endoscopic submucosal dissection was performed successfully. (d) Macroscopic finding of the specimen (H &E staining. Squared area is showing E). (e) Microscopic appearance with low‐power magnification demonstrated papillary adenocarcinoma (H &E staining. Squared area is showing F). (f) Microscopic appearance with high‐power view.

In our study, it was found that there were various differences between AIG and control groups in terms of cancer characteristics. More numbers of protruded types, larger tumor size, and histopathological papillary tumor in the upper location were observed in the AIG group (Fig. 3). More females were observed in the AIG group and it seems reasonable considering the fact of female dominancy of autoimmune diseases and AIG.^4^, ^9^

The process of cancer development in AIG is considered to be a multistage process; from hyperplasia to carcinoma via dysplasia.^15^, ^16^ While autoimmunity has been reported to be linked with gastric carcinogenesis through different pathways, direct mechanisms in AIG still remains unclear and further studies are warranted to investigate etiological contributions. In the latest study conducted in Italy,^17^ a proteomics signature of the gastric corpus in autoimmune atrophic gastritis (AAG) was identified. In AAG, it was demonstrated that the signature includes decreased abundance of proteins involved in the TCA cycle such as ATP5F1A, and increased abundance of those in structural molecule activity and cadherin binding. This study suggested a link between AAG and gastric cancer through proteomics alterations. In a case of malignant transformation of a gastric polyp in AIG, the expression of *p53* was strongly positive in the carcinoma cells and Ki67 was positive in approximately 50% of the carcinoma cells comparing to nontransformed cells.^15^ Exploring the changes of mucin phenotype during the pathological process is of great significance to reveal the occurrence and development of gastric cancer in AIG. Although our study showed distinctive features of early gastric cancer developed in AIG, further investigations are required for elucidation of the mechanism according to carcinogenesis in AIG relating to the morphological characteristics.

In this retrospective study for 5 years, we found a relatively high prevalence of AIG‐related gastric cancer (7.4%). The prevalence of AIG in patients with gastric cancer in a similar study was 4.9%.^8^ The incidence of AIG‐related gastric cancer seems to be affected by the characteristics of study cohort and endoscopists' awareness toward ‘corpus‐dominant gastritis’. The diagnostic criteria for AIG in clinical setting especially in diagnostic endoscopy have not been established. In this study, the criteria for AIG were simply defined as corpus‐predominant gastritis with serologically positive status of PCA or ICA regardless of *H. pylori* infection. AIG could also occur in *H. pylori*‐positive patients^18^, ^19^ and AIG group in our study also could have included concomitant *H. pylori*‐related gastritis. Autoimmunity might be caused and exacerbated by *H. pylori* infection, carcinogenesis, and growth mechanisms including lower mucosal acidity (pH), presence of the gastric receptor as promotion factor under hypergastrinemia, and presence or absence of apoptosis are required for evaluation. The causal effect of coexistence of *H. pylori* infection and AIG on cancer initiation and progression is still unknown. Gastric xanthoma is a representative lesion suggesting previous or ongoing *H. pylori* infection.^20^ In our study, certain numbers of gastric xanthoma were observed in *H. pylori*‐negative cases without a medical history of eradication in this study. Many cases that were previously considered as gastric cancer occurred from previous *H. pylori* infection may actually be gastric cancer associated with AIG. A causal connection between *H. pylori* infection and development of AIG have been the major focus of attention in many research studies until now^6^, ^18^, ^21^, ^22^, ^23^, ^24^, ^25^ and it is essential to elucidate causative role of each factor in cancer development.

Our study has some limitations. First, this study is a retrospective study based on a chart review in a single center and the number of AIG cases was limited. Second, the diagnostic criteria for AIG were simply defined as corpus‐predominant gastritis with serologically positive status of PCA or ICA without histopathological diagnosis that could be causing false positive cases of AIG. Although biopsy was performed in 14 out of 24 cases and histological atrophy was confirmed in our study, systematic biopsy both from the antrum and corpus is essential to prove AIG histologically. Third, all the cancer cases have not been tested for PCA and ICA. It is presumed that AIG at initial stage could have been missed and mixed into the control group due to these ambiguous criteria causing a bias between the two groups. Moreover, this study cohort focused only on early gastric cancer and advanced cancer with AIG has not been investigated. When the subjects for analysis are expanded to gastric cancers of all stages with serological examination of gastric antibodies, differences that were observed in our study may be altered. To discuss the carcinogenesis and cancer development in AIG impartially, all the stages of gastric cancer with AIG should be evaluated.

Although there are certain issues described above, our findings demonstrated unique characteristics of early gastric cancer in AIG. When gastroenterologists encounter gastric cancer lesion with these findings, it will be a good trigger to consider autoimmune disorder in the background in the era of post *H. pylori* eradication. However, predicting future cancer incidence and establishing optimal surveillance period for early detection in AIG should be inevitable issues to be handled. To overcome these issues, a proposal of efficient diagnostic criteria and algorithm for AIG not only with endoscopic appearance but also with histological and serological evidences are required for all gastritis and gastric cancer cases that are not fully covered by the medical insurance for the time being.

In conclusion, early gastric cancer associated with AIG demonstrated several different characteristics from that without AIG, including variety of tumor morphologies and histological types with female dominancy. There are still many unanswered questions that need clarification and puzzles to be solved. More cases are expected to be accumulated for further carcinogenic investigation in the near future.

### Patient and public involvement

Patients and/or the public were not involved in the design, or conduct, or reporting, or dissemination plans of this research.

## Ethics approval

The Institutional Review Board of Tokushima University Hospital provided permission for this study (No. 3480) to conduct chart reviews for each patient and informed consent was obtained from all subjects.

## Provenance and peer review

Not commissioned; externally peer reviewed.

## Data Availability

Data are available upon reasonable request to the corresponding author.
